# Hybrid of Restricted and Penalized Maximum Likelihood Method for Efficient Genome-Wide Association Study

**DOI:** 10.3390/genes11111286

**Published:** 2020-10-29

**Authors:** Wenlong Ren, Zhikai Liang, Shu He, Jing Xiao

**Affiliations:** 1 Department of Epidemiology and Medical Statistics, School of Public Health, Nantong University, Nantong 226019, China; wenlongren@ntu.edu.cn (W.R.); he_shu@ntu.edu.cn (S.H.); 2 Plant and Microbial Biology Department, University of Minnesota, Saint Paul, MN 55108, USA; liang795@umn.edu

**Keywords:** restricted maximum likelihood, penalized, computational efficiency, linear mixed model, GWAS

## Abstract

In genome-wide association studies, linear mixed models (LMMs) have been widely used to explore the molecular mechanism of complex traits. However, typical association approaches suffer from several important drawbacks: estimation of variance components in LMMs with large scale individuals is computationally slow; single-locus model is unsatisfactory to handle complex confounding and causes loss of statistical power. To address these issues, we propose an efficient two-stage method based on hybrid of restricted and penalized maximum likelihood, named HRePML. Firstly, we performed restricted maximum likelihood (REML) on single-locus LMM to remove unrelated markers, where spectral decomposition on covariance matrix was used to fast estimate variance components. Secondly, we carried out penalized maximum likelihood (PML) on multi-locus LMM for markers with reasonably large effects. To validate the effectiveness of HRePML, we conducted a series of simulation studies and real data analyses. As a result, our method always had the highest average statistical power compared with multi-locus mixed-model (MLMM), fixed and random model circulating probability unification (FarmCPU), and genome-wide efficient mixed model association (GEMMA). More importantly, HRePML can provide higher accuracy estimation of marker effects. HRePML also identifies 41 previous reported genes associated with development traits in *Arabidopsis*, which is more than was detected by the other methods.

## 1. Introduction

Genome-wide association studies (GWAS) can advance our understanding of molecular mechanism of complex traits [1,2,3,4]. Testing each SNP (single nucleotide polymorphism) one time is the most popular method, which is flexible to perform on all kinds of models. However, each SNP requires multiple testing adjustment, which will result in strict *p*-values. One strategy to solve this problem is to use more information beyond the *p*-value. For example, Xu, et al. [5] proposed a model-based clustering method that borrowed information across SNPs and increased the signal strength by properly clustering SNPs. Lee and Lee [6] presented a web application for the network-based *Arabidopsis* genome-wide association boosting, which can identify weak association signals by integrating co-functional gene network information. Apart from this, the linear mixed model (LMM) has become a widely used methodology due to its capability in controlling for population stratification and the inclusion of related individuals [7]. However, the implementation of LMM requires estimating the variance component of each random effect, leading to increased computational burden. The restricted maximum likelihood (REML) is the widely used method for the estimation of variance components. Conventional REML algorithms are impractical to handle large-scale genomic datasets with thousands of individuals and millions of SNPs. There are two main reasons limiting REML application: on one hand, it is hard to obtain closed-form solutions of REML or posterior estimations of variance components. On the other hand, the inversion of the covariance matrix is required to perform on each computation of likelihood, an operation that is proportional to the cube of individual number. As a result, improving the computational efficiency of REML for estimating variance components has become one of the research hotspots [8,9,10,11,12].

With regards to this, several approaches based on sparse matrix operations have been developed to improve the calculating speed [13,14]. Lippert, et al. [8] performed spectral decomposition on the covariance matrix, converting matrix inversion to diagonal reciprocal operation. This strategy not only greatly improves the computational efficiency but, also, takes advantage of genetic relatedness matrix to adjust the correlation. Similarly, Zhou and Stephens [9] implemented their method in a genome-wide efficient mixed model association (GEMMA), which only required a small amount of matrix vector multiplications to obtain variance components. A different idea was proposed by Loh et al. [10], which used preconditioned conjugate gradients and stochastic trace estimators to avoid all cubic operations. This is an asymptotic method via linear system transformations, particularly suitable for Bio Bank large-scale individuals. In addition to the above two popular ideas, some specialized methods have been developed to solve variance component estimations efficiently. Lourenco et al. [15] proposed a robust derivative-free restricted-maximum likelihood framework (DF-REML), which can tackle normality violations, as well as other model misspecifications. Cesarani et al. [16] investigated bias in variance components under different genotyping strategies, showing that single-step genomic restricted maximum likelihood (ssGREML) is more robust compared to GREML. Ganjgahi et al. [4] proposed a weighted least squares REML (WLS-REML) using a noniterative one-step random effect estimator to decrease the computational cost. Border and Becker [12] developed stochastic *Lanczos* derivative-free REML and *Lanczos* first-order Monte Carlo REML to further improve the computing speed. However, these existing methods for REML variance components estimation are mainly aimed at single-locus LMM, which is not effective enough to handle a complex genetic structure.

Several classical multivariate selection methods have good performances in association analyses when the number of SNPs is not far more than that of individuals, including Lasso and its derivatives [17,18,19], penalized maximum likelihood (PML) [20,21,22,23], and Bayesian methods [24,25]. However, most of these methods are not available to analyze large-scale genomic data due to ultra-high dimensional variables. To address this issue effectively, some improved multi-locus GWAS methods were proposed. For example, multi-locus mixed-model (MLMM) [26] adopts stepwise mixed-model regression with forward inclusion and backward elimination using a Bayesian approach and performs well when the structure is complex, fixed and random model circulating probability unification (FarmCPU) [27] incorporates multiple markers simultaneously as covariates in a stepwise LMM to partially remove the confounding between testing markers and kinship, iterative nonlocal prior-based selection (GWASinlps) [28] considers an iterative structured screen-and-select strategy and nonlocal priors within it and provides an efficient and parsimonious variable selection for continuous phenotypes, a machine-learning method combines a random forest-based technique with the modeling of linkage disequilibrium through latent variables [29] and accelerates the computing speed for multi-locus GWAS, a gene set analysis with Generalized Berk-Jones (GBJ) statistic [30] introduces a permutation-free parametric framework, which can increase the power by incorporating information from multiple signals in the same gene, the SNP set GWAS approach RAINBOW [31] achieves faster computation by using linear kernel for constructing the Gram matrix of the SNP set of interest, and the multi-locus random SNP effect mixed linear model (mrMLM) [32] uses the Wald test based on a random SNP effect linear mixed model to reduce dimensionality; then, all the selected markers are placed into an empirical Bayes [33] multi-locus model, showing the advantage in controlling a complex population structure. A limitation of Bayesian method is that Markov Chain Monte Carlo (MCMC) sampling comes at the cost of intensive computation, or the posterior distribution of fitness is not easy to calculate [34]. Penalized maximum likelihood is similar to the Bayesian method involving the posterior distribution of parameters; the difference is that PML adopts a fast approach to obtain the maximum posteriori estimates of fitness via numerical optimization. Therefore, PML provides an efficient way to perform multivariate selection.

In this study, we developed an efficiently hybrid method HRePML to perform GWAS, which takes full advantage of REML and PML. Under the linear mixed model framework, we firstly conducted single-locus marker scanning using REML and then carried out multi-locus marker selection based on the reduced subset, taking genetic relatedness and population stratification into account. We used pure C++ language to implement HRePML and overcome one key issue in the programming limited memory Broyden–Fletcher–Goldfarb–Shanno (BFGS) method [35]. In order to validate the effectiveness of HRePML, we conducted a series of simulation studies and real data analyses and compared it with three methods: MLMM [26], FarmCPU [27], and GEMMA [9].

## 2. Materials and Methods

### 2.1. The Arabidopsis thaliana Dataset

A publicly available dataset of *Arabidopsis thaliana* [36] is used to conduct a simulation study and real data analysis. This dataset contains 216,130 markers and 199 individuals. There are four development related traits to be analyzed, which are the number of days between the appearance of the first flower and the senescence of the last flower (FT duration GH), number of days between germination and plant complete senescence (LC duration GH), number of days between germination and senescence of the last flower (LFS GH), and number of days between last flower senescence and complete plant senescence (MT GH), respectively.

### 2.2. Restricted Maximum Likelihood (REML) Method in Single-Locus Screening Stage

#### 2.2.1. Single-Locus Linear Mixed Model

A standard linear mixed model for association mapping can be expressed as

(1) y=Fb+Xβ+u+ε

where y denotes the n×1 observed phenotypic vector of n individuals, F is an n×c fixed effect design matrix, including 1′s column vector, b is a c×1 vector of their corresponding effect sizes, X denotes the n×1 marker genotype vector of focal variant, β is the random effect of one focal marker with normal distribution $\beta ∼N(0,\sigma_{g}^{2})$, the variance $\sigma_{g}^{2}$ is changed with different markers, and $u∼N(0,\sigma_{u}^{2}\Sigma_{u})$ is an n×1 random vector and is typically used to account for polygenic effects or confounding factors; here, $\sigma_{u}^{2}$ is the variance, and $\Sigma_{u}$ is an n×n covariance structure defined as $\Sigma_{u}=G\cdot G^{T}/m$; G is an n×m genotype matrix, and m is the number of markers; $\varepsilon ∼Ν(0,\sigma_{n}^{2}{Ι}_{n})$ denotes an n×1 independently and identically distributed (i.i.d.) residual vector, and $\sigma_{n}^{2}$ is the residual variance. The covariance of y can be denoted as

(2) $$\begin{array}{cc} Cov(y)= & XX^{T}\sigma_{g}^{2}+\Sigma_{u}\sigma_{u}^{2}+I_{n}\sigma_{n}^{2}\\ = & \left[XX^{T}\omega_{g}+\left(\Sigma_{u}\omega_{u}+I_{n}\right)\right]\sigma_{n}^{2}\\ = & \left(XX^{T}\omega_{g}+P\right)\sigma_{n}^{2} \end{array}$$

where $\omega_{g}=\sigma_{g}^{2}/\sigma_{n}^{2}$, $\omega_{u}=\sigma_{u}^{2}/\sigma_{n}^{2}$, and $P=\Sigma_{u}\omega_{u}+I_{n}$. The estimate of $\omega_{u}$ can be obtained under the null model, defined as ${\hat{\omega }}_{u}$, which only needs to be computed once. Using spectral decomposition, $\Sigma_{u}$ can be expressed as $\Sigma_{u}=Q\Lambda Q^{T}$, where $\Lambda =diag(\lambda_{1},\lambda_{2},\cdots ,\lambda_{n})$ is a diagonal matrix of eigenvalues, and Q is an n×n eigenvector matrix corresponding to these eigenvalues [32,37,38].

#### 2.2.2. Equation Transformation and Update Covariance

Transform Equation (1) by left-multiplying $Q^{T}$ [32] and generate the following model

(3) $$y_{Q}=F_{Q}b+X_{Q}\beta +Q^{T}u+Q^{T}\varepsilon$$

where $y_{Q}=Q^{T}y$, $F_{Q}=Q^{T}F$, and $X_{Q}=Q^{T}X$. After transformation, the covariance of *y* becomes

(4) $$\begin{array}{cc} Cov(y_{Q})= & X_{Q}X_{Q}^{T}\sigma_{g}^{2}+Q^{T}\Sigma_{u}\sigma_{u}^{2}Q+Q^{T}I_{n}\sigma_{n}^{2}Q\\ = & X_{Q}X_{Q}^{T}\sigma_{g}^{2}+Q^{T}Q\Lambda Q^{T}Q\sigma_{u}^{2}+Q^{T}I_{n}Q\sigma_{n}^{2}\\ = & X_{Q}X_{Q}^{T}\sigma_{g}^{2}+\Lambda \sigma_{u}^{2}+I_{n}\sigma_{n}^{2}\\ = & \left(X_{Q}X_{Q}^{T}\omega_{g}+\Lambda {\hat{\omega }}_{u}+I_{n}\right)\sigma_{n}^{2} \end{array}$$

Let $V_{0}=\Lambda {\hat{\omega }}_{u}+I_{n}$ and $V=X_{Q}X_{Q}^{T}\omega_{g}+V_{0}$; clearly, diagonal matrix $V_{0}$ is estimated. To determine whether a marker has an effect, hypothesis testing needs to be conducted. To estimate the marker effect β, its variance ratio $\omega_{g}$ needs to be obtained firstly, so that the estimation of each $\omega_{g}$ is the most interesting issue for each corresponding marker.

#### 2.2.3. Optimal Solution via Efficient REML

In order to get the estimation ${\hat{\omega }}_{g}$, optimize the following restricted log-likelihood function with respect to $\omega_{g}$,

(5) $$\begin{array}{cc} L_{r}(\omega_{g})= & -\frac{1}{2}\log\left|V\right|-\frac{1}{2}\log\left|F_{Q}^{T}V^{-1}F_{Q}\right|-\frac{n-c}{2}{\left(y_{Q}-F_{Q}\hat{b}\right)}^{T}V^{-1}\left(y_{Q}-F_{Q}\hat{b}\right)\\ = & -\frac{1}{2}\log\left|V\right|-\frac{1}{2}\log\left|F_{Q}^{T}V^{-1}F_{Q}\right|-\frac{n-c}{2}y_{Q}^{T}Sy_{Q} \end{array}$$

where

(6) $$\hat{b}={\left(F_{Q}^{T}V^{-1}F_{Q}\right)}^{-1}F_{Q}^{T}V^{-1}y_{Q}$$

(7) $$S=V^{-1}-V^{-1}F_{Q}{\left(F_{Q}^{T}V^{-1}F_{Q}\right)}^{-1}F_{Q}^{T}V^{-1}$$

Limited-memory BFGS (L-BFGS) [35,39] is an optimized algorithm of quasi-Newton methods for efficient solution. The libLBFGS is a user-friendly C library implementation of the L-BFGS method written by Nocedal [40]. This library requires that the objective function $L_{r}(\omega_{g})$ and its gradient $\partial L_{r}(\omega_{g})/\partial \omega_{g}$ are computable. However, the gradient function cannot be obtained directly by closed form. Fortunately, the well-known C++ Boost library [41] provides a finite difference method for solving the gradient, which is located in a boost/math/differentiation/finite_difference.hpp file. After ${\hat{\omega }}_{g}$ is estimated, $\hat{\beta }$ and ${\hat{\sigma }}_{n}^{2}$ can be easily obtained for restricted log-likelihood functions, which are as follows, respectively,

(8) $$\hat{\beta }=\omega_{g}X_{Q}^{T}Sy_{Q}$$

(9) $${\hat{\sigma }}_{n}^{2}=\frac{1}{n-c}y_{Q}Sy_{Q}$$

so that the variance of $\hat{\beta }$ denotes

(10) $$\mathrm{var}(\hat{\beta })=\omega_{g}{\hat{\sigma }}_{n}^{2}-\omega_{g}X_{Q}^{T}V^{-1}X_{Q}\omega_{g}{\hat{\sigma }}_{n}^{2}$$

The Wald test is used to conduct a hypothesis test on each marker effect β—that is, $H_{0}:\beta =0,H_{1}:\beta \neq 0$. The Wald test is

(11) $$W=\frac{{\hat{\beta }}^{2}}{\mathrm{var}(\hat{\beta })}$$

W follows the chi-square distribution with 1 degree of freedom, and the *p*-value corresponding to W can be computed by the C++ Boost library, which is located in a boost/math/distributions/chi_squared.hpp file. In the single-marker screening stage, a relatively loose and flexible *P* cutoff is adopted.

### 2.3. Penalized Maximum Likelihood (PML) Method in Multi-Locus Screening Stage

#### 2.3.1. Multi-Locus Linear Mixed Model and Penalized Likelihood Function

All the markers passing the REML step are placed into a multi-locus model [20] as

(12) $$y=Fb+\sum_{i=1}^{t}x_{i}\beta_{i}+\varepsilon$$

where y, F, b, and ε are the same definitions as Equation (1). $x_{i}$ is the *i*th n×1 genotypic vector, and $\beta_{i}$ is the fixed effect of corresponding marker. t denotes the total number of selective markers from the REML step. 

Penalized maximum likelihood (PML) is a fast approach to use numerical optimization to obtain the maximum posteriori estimates when the penalty function is a probability density on the parameters [20,22,42]. Let $\theta =\left\{b,\beta_{1},\cdots ,\beta_{t},\sigma_{n}^{2}\right\}$ is the interesting vector of the parameters. The log-likelihood function denotes

(13) $$L(\theta )=\log\varphi \left(y;Fb+\sum_{i=1}^{t}x_{i}\beta_{i},I\sigma_{n}^{2}\right)$$

where $\varphi \left(y;Fb+\sum_{i=1}^{t}x_{i}\beta_{i},I\sigma_{n}^{2}\right)$ is the normal density with the mean $Fb+\sum_{i=1}^{t}x_{i}\beta_{i}$ and covariance $I\sigma_{n}^{2}$. A factor is introduced to penalize on the marker effects $\beta_{i}$

(14) $$p(\beta_{i})=\varphi \left(\beta_{i};\mu_{i},\sigma_{i}^{2}\right)\;\;\;\;\;i=1,\cdots ,t$$

where $\varphi \left(\beta_{i};\mu_{i},\sigma_{i}^{2}\right)$ is a normal prior with a mean $\mu_{i}$ and variance $\sigma_{i}^{2}$. Then, $\mu_{i}$ is also assigned a normal prior distribution

(15) $$p(\mu_{i})=\varphi \left(\mu_{i};0,\sigma_{i}^{2}/\tau \right)\;\;\;\;\;i=1,\cdots ,t;\tau >0$$

Let $\delta =\left\{\mu_{1},\cdots ,\mu_{t},\sigma_{1}^{2},\cdots ,\sigma_{t}^{2}\right\}$ be the hyperparameters that can be estimated along with the interested parameters at the same time. The logarithm of the penalized prior distribution is

(16) $$P\left(\theta ,\delta \right)=\sum_{i=1}^{t}\left[\log\varphi \left(\beta_{i};\mu_{i},\sigma_{i}^{2}\right)+\log\varphi \left(\mu_{i};0,\sigma_{i}^{2}/\tau \right)\right]$$

Then, the logarithm of penalized likelihood function can be defined as

(17) $$L_{p}\left(\theta ,\delta \right)=L\left(\theta \right)+P\left(\theta ,\delta \right)$$

#### 2.3.2. Iterative Method for Parameter Estimation

The parameter vector θ and δ are estimated by the penalized maximum likelihood simultaneously, and the solution of PML needs an iterative method to implement. For the interested parameter vector θ, find the first-order partial derivatives of the elements in θ and then make them equal to 0.

Via $\frac{\partial }{\partial b}L_{p}\left(\theta ,\delta \right)=0$, $\frac{\partial }{\partial \beta_{i}}L_{p}\left(\theta ,\delta \right)=0$, and $\frac{\partial }{\partial \sigma_{n}^{2}}L_{p}\left(\theta ,\delta \right)=0$ and i=1,⋯,t, it can obtain a closed-form expression on b, $\beta_{i}$, and $\sigma_{i}^{2}$, respectively.

(18) $$b={\left(F^{T}F\right)}^{-1}F^{T}\left(y-\sum_{i=1}^{t}x_{i}\beta_{i}\right)$$

(19) $$\beta_{i}={\left[x_{i}^{T}x_{i}+\sigma_{n}^{2}/\sigma_{i}^{2}\right]}^{-1}\left[x_{i}^{T}\left(y-Fb-\sum_{\begin{array}{l} k=1\\ k\neq i \end{array}}^{t}x_{k}\beta_{k}\right)+\mu_{i}\sigma_{n}^{2}/\sigma_{i}^{2}\right]$$

(20) $$\sigma_{n}^{2}=\frac{1}{n}{\left(y-Fb-\sum_{i=1}^{t}x_{i}\beta_{i}\right)}^{T}\left(y-Fb-\sum_{i=1}^{t}x_{i}\beta_{i}\right)$$

For the nuisance parameter vectors, δ, $\mu_{i}$, and $\sigma_{i}^{2}$ are acquired in the same way. Via $\frac{\partial }{\partial \mu_{i}}L_{p}\left(\theta ,\delta \right)=0$ and $\frac{\partial }{\partial \sigma_{i}^{2}}L_{p}\left(\theta ,\delta \right)=0$, $\mu_{i}=\beta_{i}/\left(\tau +1\right)$ and $\sigma_{i}^{2}=\frac{1}{2}\left[{\left(\beta_{i}-\mu_{i}\right)}^{2}+\tau \mu_{i}^{2}\right]$ can be obtained.

Set the initial values for θ, δ, and τ and update the values of b, $\beta_{i}$, $\sigma_{n}^{2}$, $\mu_{i}$, and $\sigma_{i}^{2}$ until convergence. In order to reach convergence quickly, a criterion according to the experience needs to be considered. After the parameter estimation, $\left|\beta_{i}\right|>10^{-4}$ are selected to further conduct a likelihood-ratio test. The logarithm of the odds (LOD) score is used to determine the final identification.

### 2.4. Design of Simulation Experiments

Four simulation experiments were designed to validate our new method HRePML. In the first simulation study, our goal was to explore the new method’s performance on the statistical power, mean square error (MSE), and running time. We generated a set of genotype data consisting of 500 individuals and 10,000 markers, which was based on the *Arabidopsis thaliana* dataset [36]. Eight quantitative trait nucleotides (QTNs) were simulated with heritability of 0.01, 0.03, 0.03, 0.05, 0.08, 0.01, 0.05, and 0.05, respectively. Their positions and true effects are described in Table 1. The total genetic heritability is $h_{T}^{2}=\sigma_{G}^{2}/(\sigma_{G}^{2}+\sigma_{e}^{2})=0.01\times 2+0.03\times 2+0.05\times 3+0.08=0.31$, and the residual variance is $\sigma_{e}^{2}=10.0$. Then, the total genetic variance $\sigma_{G}^{2}$ can be obtained, as well as the genetic variance of each QTN $\sigma_{gi}^{2}(i=1,\cdots ,8)$. The population mean is set to 10.0. The phenotype is generated by the model $y=\mu +\sum_{i=1}^{8}x_{i}\beta_{i}+\varepsilon$, where $\varepsilon \sim MVN(0,10.0\times I_{n})$. The experiment is repeated 1000 times. The statistical power for each QTN is defined as the percentage of the number of detected QTN to the number of repetitions. We used the logarithm of the odds (LOD = 3.0) as the criterion for detecting QTN [32,43,44]. Mean squared error was calculated as $MSE_{k}=\frac{1}{S}\sum_{i=1}^{S}{\left({\hat{\beta }}_{ki}-\beta_{k}\right)}^{2}$, where k=1,⋯,8, S was the number of detected QTN *k* among 1000 repetitions, ${\hat{\beta }}_{ki}$ was the effect estimation of QTN *k* in the ith repeat, and $\beta_{k}$ was the *k*th QTN’s true effect. 

In the second simulation study, we aimed at exploring the influence of polygenic background on HRePML. The polygenic effects were introduced with multivariate normal distribution $MVN(0,\sigma_{pg}^{2}K)$, where the polygenic variance $\sigma_{pg}^{2}$ was set to 2.0, genetic relatedness matrix K was calculated as $G^{T}G/M$, G was the genotype matrix, and M was the number of markers. Other parameters were set to the same as the first simulation study. The position and true effect of each QTN are listed in Table S1. Based on the model $y=\mu +\sum_{i=1}^{8}x_{i}\beta_{i}+u+\varepsilon$, where u~ΜVN(0,2.0×K), the phenotypes are simulated. 

In the third simulation study, our goal was to investigate the influence of the sample size on the running time and statistical power. The sample size was set to 500, 1000, 2000, and 4000, respectively. Meanwhile, the number of markers was fixed at 10,000. In the fourth simulation study, our aim was to investigate the impact on running time as the number of markers increased. The number of markers was set to 10,000, 50,000, 100,000, and 200,000, respectively. At the same time, the sample size was fixed at 500. In these two simulation studies, the repeat times were set to 100. The position and heritability of each QTN were set to the same as those of first simulation study. Their parameters are listed in Table S3.

## 3. Results

### 3.1. Statistical Properties

**Figure 1:**
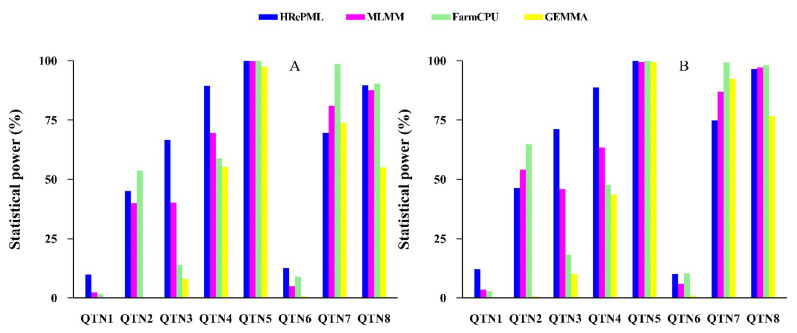
Comparison of statistical powers of eight simulated quantitative trait nucleotides (QTNs) using four genome-wide association study (GWAS) methods (hybrid of restricted and penalized maximum likelihood (HRePML), multi-locus mixed-model (MLMM), fixed and random model circulating probability unification (FarmCPU), and genome-wide efficient mixed model association (GEMMA)). (**A**) The first simulation study: no polygenic background. (**B**) The second simulation study: an additive polygenic variance involved.

**Figure 2:**
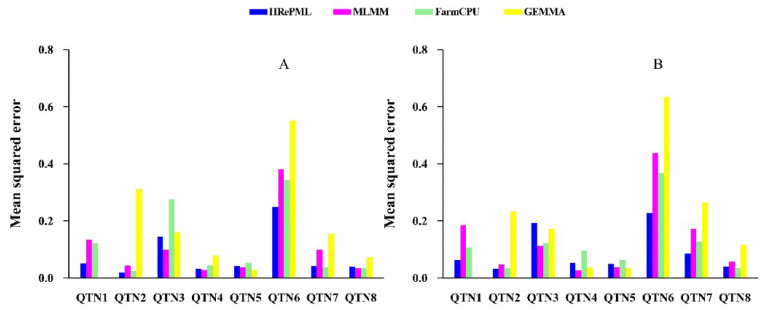
Comparison of mean squared errors of each simulated QTN effect using four GWAS methods (HRePML, MLMM, FarmCPU, and GEMMA). The descriptions in (**A**,**B**) are the same as those in Figure 1.

We compared the statistical properties of the new HRePML method with those of the multi-locus mixed-model (MLMM) [26], fixed and random model circulating probability unification (FarmCPU) [27], and genome-wide efficient mixed model association (GEMMA) [9] methods. Here, the statistical properties mainly included statistical power and mean squared error (MSE). In the first simulation study, the dataset consisted of 500 individuals and 10,000 SNP markers with 1000 replicates. Eight true QTNs were set in each replicate. Then, this dataset was regarded as having 10,000,000 SNPs and 8000 true QTNs in total. The average power of the four methods were 60.39%, 53.15%, 53.21%, and 36.34%, respectively. HRePML obtained the highest statistical power, which was at least about 7% higher than the other three methods. In particular, HRePML performed well on QTNs with lower heritability, such as QTN 1, 3, 4, and 6 (Table 1 and Table 2 and Figure 1A). The mean squared error was used to measure the accuracy of the QTN effect estimates, and smaller MSE represented better accuracy. The average MSE of the above four methods were 0.0772, 0.1068, 0.1165, and 0.1933, respectively, demonstrating that the average MSE of HRePML was the minimum (Table 1 and Table 2 and Figure 2A).

To further validate the performance of HRePML, an additive polygenic effect was involved in the second simulation study. The dataset was the same with that used in the first simulation study, except that polygenic effect was added to the phenotype. The same trend in statistical power was observed, and the average powers of HRePML, MLMM, FarmCPU, and GEMMA were 62.45%, 57.08%, 55.18%, and 40.46%, respectively, which showed that HRePML was still powerful and robust under polygenic interference (Tables S1 and S2 and Figure 1B). As far as the mean squared error was concerned, the average MSE of the above four methods were 0.0926, 0.1343, 0.1184, and 0.2125, respectively. HRePML had the most accuracy of the QTN effect estimates, followed by FarmCPU, MLMM, and GEMMA (Tables S1 and S2 and Figure 2B).

**Figure 3:**
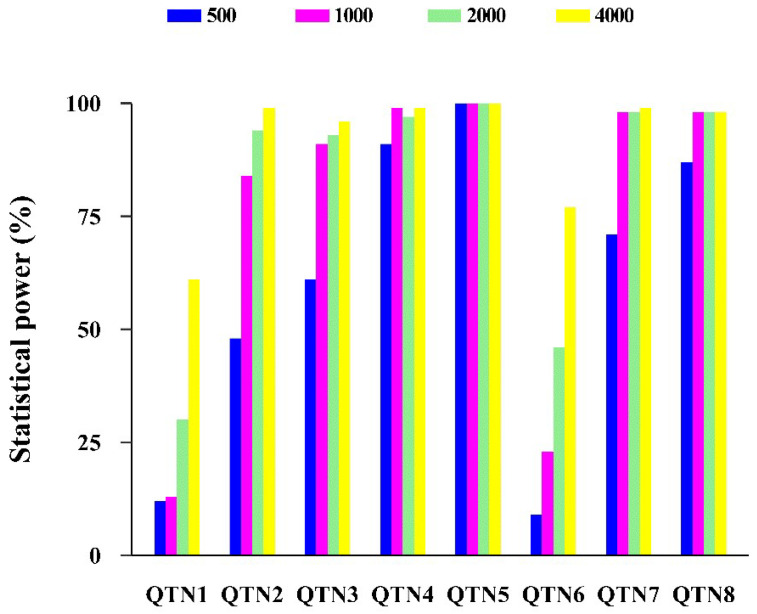
Effect of the sample size on the statistical power using HRePML in the third simulation study.

In the third simulation study, we investigated the effect of the sample size on the statistical power of HRePML. There were four datasets consisting of 500, 1000, 2000, and 4000 individuals, respectively, and 10,000 SNP markers, with 100 replicates. Eight true QTNs were set in each replicate. Then, each dataset could be regarded as having 1,000,000 SNPs and 800 true QTNs in total. The average powers of sample sizes 500, 1000, 2000, and 4000 were 59.88%, 75.75%, 82.00%, and 91.13%, respectively. Clearly, the statistical power improved as the sample size increased. The results demonstrated that the statistical power could be more than 80% for QTN, with heritability equal or greater than 0.03 when the sample size reached 1000. However, for QTN with very small heritability (0.01), the required sample size was at least 4000, and then, the statistical power could exceed 60% (Table 3 and Figure 3).

### 3.2. Running Time

**Figure 4:**
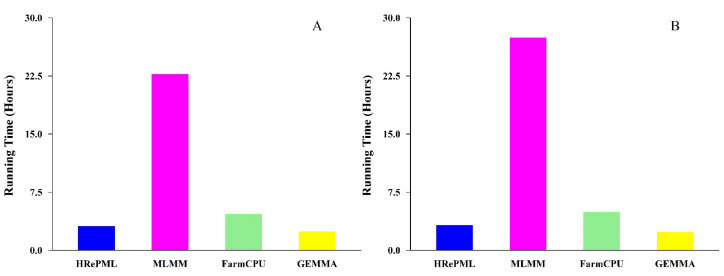
Comparison of total running time using four GWAS methods (HRePML, MLMM, FarmCPU, and GEMMA). The descriptions in (**A**,**B**) are the same as those in Figure 1.

**Figure 5:**
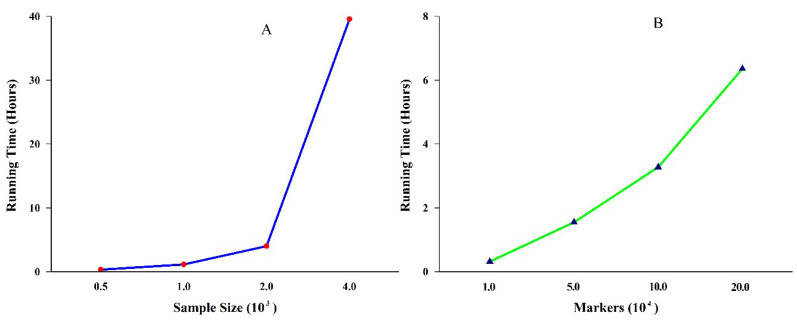
(**A**). Effect of the sample size on running time in the third simulation. (**B**) Effect of markers on the running time in the fourth simulation.

All above four methods were carried out on the same machine (Intel^®^ Core™ i5-7300HQ CPU 2.50 GHz, Memory 8.00 GB, Houston, TX, USA). In the first simulation consisting of 1000 repetitions, the total running time of HRePML, MLMM, FarmCPU, and GEMMA were 3.1419, 22.7274, 4.6653, and 2.4186 h, respectively. Compared with the other two multi-locus MLMM and FarmCPU methods, HRePML was the most computationally efficient, which was only slightly slower than the single-locus GEMMA method. In particular, HRePML achieved about seven times faster than the popular multi-locus method MLMM (Table 2 and Figure 4A). The second simulation also conducted with 1000 repetitions, and the same trend in total running time was observed, which was 3.2273, 27.4473, 4.9198, and 2.3855 h for the four methods, respectively. GEMMA was the fastest method, followed by HRePML, FarmCPU, and MLMM (Table S2 and Figure 4B). In the third and fourth simulations of 100 repeated experiments on the HRePML, the sample sizes and number of markers were investigated on the influence of running time. With sample sizes 500, 1000, 2000, and 4000, the running times were 0.3142, 1.1244, 3.9969, and 39.5439 h, respectively. The results showed that, as the sample size increased, the running time increased nonlinearly (Table 3 and Figure 5A). In the fourth simulation study, there were four datasets consisting of 10,000, 50,000, 100,000, and 200,000 SNP markers, respectively, and 500 individuals. With the number of markers 10,000, 50,000, 100,000, and 200,000, the running time was 0.3142, 1.5460, 3.2735, and 6.3574 h, respectively. Clearly, the running time increased almost linearly with the markers increasing (Figure 5B).

### 3.3. Association Analysis of Real Data in Arabidopsis

We performed GWAS on four development traits of *Arabidopsis* using HRePML, MLMM, FarmCPU, and GEMMA. The four methods identified 77, 43, 32, and 17 SNPs significantly associated with four traits. HRePML had the highest number of detected SNPs, which was more than four times than that of GEMMA detected (Table S4). Then, we performed gene ontology (GO) functional annotations on detected SNPs within their physical position 10 KB. As a result, the number of candidate genes detected by four methods were 41, 19, 25, and 5, which demonstrated that HRePML had the strongest ability to mine candidate genes, followed by FarmCPU, MLMM, and GEMMA (Tables S4 and S5). A total of eight genes were detected simultaneously by at least two methods. Interestingly, most of these eight genes were located on chromosome 5, while there was none located on chromosome 1. We found good agreement between the new methods HRePML and FarmCPU. It was worth noting that *AT5G45900* and *AT5G45940* could be identified by at least two methods on traits LC duration GH and LFS GH (Table 4). *AT5G45900* is a component of the autophagy conjugation pathway and contributes to plant basal immunity towards fungal infection. *AT5G45940* encodes a CoA pyrophosphatase and also has ppGpp pyrophosphohydrolase and exhibits minor activity of NADH pyrophosphatase and was most strongly expressed in embryo cotyledon and the hypocotyl, flower, and phloem of vascular tissues [45]. In summary, HRePML identified the most numbers of significantly associated SNPs and genes in the real data analysis (Table S5).

### 3.4. An Example for the Use of HRePML

To run HRePML requires four input files. The first input file is a genotype file, where each row represents the SNP marker, and each column represents an individual. The first two columns of the genotype file provide the chromosome and physical position information about the SNP marker. The genotype is coded as 0, 1, and 2, representing aa, Aa, and AA, where “A” is a dominant allele and “a” is a recessive allele. The second input file is the phenotype file, which is a column vector. The third input file is the genetic relatedness matrix or kinship file, which is a N×N matrix, and N is the number of individuals. The fourth input file is the covariates file, where the first column is the unit column vector, followed by the population structure or principal component matrix. The example data can be found at https://github.com/wenlongren/HRePML/tree/master/Example%20Data.

In a Linux system (Ubuntu), the compiling command is: g++ -I/path/liblbfgs-1.10/include HRePML-Linux.cpp -llapack -lblas -llbfgs -o output, where it needs to include the C++ library of limited-memory BFGS. If Math Kernel Library (MKL) has been installed for Intel CPU users, the following compiling command is recommended: g++ -I/path/liblbfgs-1.10/include HRePML-Linux.cpp -lmkl_gf_lp64 -lmkl_sequential -lmkl_core -llbfgs -o output. In order to save the results, the HRePML program requires two output files, which are the results file and the computational time file. After compilation, the execution command is: ./output Genotype.csv Phenotype.csv Kinship.csv Fixed.csv Results.csv Time.csv. Then, the results and running time are output into Results.csv and Time.csv files, respectively.

## 4. Discussion

In this paper, we proposed a new fast multi-locus method HRePML for GWAS, which is based on a restricted and penalized maximum likelihood function. HRePML can take genetic relatedness and population stratification into account under the linear mixed model. In addition, we implemented the algorithm in pure C++ language and provide Windows and Linux platform versions for the researcher’s choice.

The new method adopts a two-stage approach to conduct multi-locus GWAS, which is widely used to improve the computational efficiency when hundreds of thousands of SNPs appear. The core idea is to conduct an initial screening of the marginal effects of all SNPs and select the ones with reasonably large effects for the next phase of the multi-locus test. Recently, multi-locus GWAS methods have become more and more popular, such as, MLMM [26], FarmCPU [27], mrMLM [32], pKWmEB (integration of Kruskal-Wallis test with empirical Bayes with polygenic background control) [43], and ISIS EM-BLASSO (iterative modified-sure independence screening and expectation-maximization bayesian least absolute shrinkage and selection operator) [44]. We drew on their successful experience and used the LOD value instead of the *p* value to determine the final identified SNPs. Using LOD equal to 3.0 as the threshold, many real data analyses show that it is feasible to improve the statistical power [32,43,44]. However, these multi-locus methods mentioned above are all programmed in R language, which are limited in analyzing large samples and massive SNP data. We implemented a new method HRePML in pure C++ language with the aid of the lapack, blas, libfgs, and boost C++ library. More importantly, the HRePML program can be further sped up with math kernel library (MKL) for Intel CPU users. Our first and second simulation experiments indicated that HRePML is about seven times faster than MLMM (Table 2 and Table S2 and Figure 4). 

HRePML can be flexibly applied to animal and human GWAS, not limited to plant research. Genetic architecture is more complex, and the genome is much larger in animals and human beings than that in plants. One important issue is allelic heterogeneity, which cannot be effectively handled by traditional single-locus methods. More importantly, genetic heterogeneity can lead to a noncausative marker being a better descriptor of the phenotype than a causative one [46]. Another common issue is rare variant architecture, which may not always be resolved by increasing the sample size. HRePML, as one multi-locus method, can consider the complex genetic architecture and deal with these two issues well. Although the current version HRePML can analyze large samples with quantitative traits in humans, animals, or plants (Figure 5), it is not available to the UK BioBank scale data [47]. We recommend BOLT-LMM [10] for analyzing biobank scale samples.

The current study in *Arabidopsis* real data analysis showed that the results have relatively low consistency among HRePML, MLMM, FarmCPU, and GEMMA (Table 4 and Table S5). There are several possible reasons for this phenomenon. Firstly, the genetic structure of real data is more complex compared with simulated data, and large errors exist in phenotypic measurements. This can lead to reduce statistical power. Secondly, different methods are based on different assumptions and different models. The first three methods adopted a multi-locus model, while GEMMA used a single-locus model. Besides that, HRePML and MLMM were based on infinitesimal genetic architectures under a linear mixed model, while FarmCPU iterated on a fixed model and a random model. Thirdly, different methods respond differently to the effects of sample size, marker numbers, allele frequency, and heritability. In our opinion, there is complementarity between the various methods, and real data analysis requires considering the results of several methods together.

## 5. Conclusions

We proposed an alternative for fast multi-locus GWAS, based on the integration of the restricted and penalized maximum likelihood. Both the simulated and real data analyses demonstrated that our method HRePML improved the statistical power significantly compared with MLMM, FarmCPU, and GEMMA. In addition, HRePML can provide a higher accuracy estimation of the marker effects. More importantly, we developed an efficient tool in pure C++ for the Windows and Linux platform. With the aid of the optimized math kernel library (MKL), HRePML can compute more efficiently when handling large individuals and millions of markers.

## Figures and Tables

**Table 1:** Comparison of the statistical power and mean squared errors (MSE) for each quantitative trait nucleotide (QTN) among the hybrid of restricted and penalized maximum likelihood (HRePML), multi-locus mixed-model (MLMM), fixed and random model circulating probability unification (FarmCPU), and genome-wide efficient mixed model association (GEMMA) methods in the first simulation study *.

QTN	Chr.	Position(bp)	R^2^	Effect	Power (%)				Mean Squared Errors (MSE)			
					HRePML	MLMM	FarmCPU	GEMMA	HRePML	MLMM	FarmCPU	GEMMA
1	1	404108	0.01	0.4328	9.9	2.4	1.6	0.0	0.0509	0.1334	0.1224	na ^#^
2	1	636788	0.03	0.7497	45.1	39.9	53.7	0.2	0.0193	0.0440	0.0241	0.3112
3	3	507976	0.03	0.7497	66.7	40.1	13.9	8.1	0.1443	0.0992	0.2756	0.1597
4	3	931437	0.05	0.9679	89.5	69.5	58.8	55.3	0.0321	0.0276	0.0434	0.0770
5	4	75898	0.08	1.2243	100.0	99.8	100.0	97.5	0.0407	0.0375	0.0527	0.0283
6	4	461978	0.01	0.4328	12.7	5.0	8.9	0.7	0.2488	0.3808	0.3429	0.5502
7	4	607026	0.05	0.9679	69.6	80.9	98.5	73.8	0.0421	0.0988	0.0367	0.1544
8	5	282008	0.05	0.9679	89.6	87.6	90.3	55.1	0.0397	0.0334	0.0345	0.0725

* In the first simulation study, the dataset consists of 500 individuals and 10,000 single nucleotide polymorphism (SNP) markers with 1000 replicates. Eight true QTNs are set in each replicate. Then, this dataset can be regarded as having 10,000,000 SNPs and 8000 true QTNs in total. ^#^ “na” represents not available.

**Table 2:** Comparison of average statistical power, average mean squared errors (MSE), and running time among the HRePML, MLMM, FarmCPU, and GEMMA methods in the first simulation study *.

Statistical Properties	HRePML	MLMM	FarmCPU	GEMMA
Average power (%)	60.39	53.15	53.21	36.34
Average MSE	0.0772	0.1068	0.1165	0.1933
Running time (Hour)	3.1419	22.7274	4.6653	2.4186

* The dataset used in Table 2 is the same as that used in Table 1.

**Table 3:** Effect of the sample size on the statistical power and running time using the HRePML method in the third simulation study *.

QTN	R^2^	Sample Size: Power (%)			
		500	1000	2000	4000
1	0.01	12	13	30	61
2	0.03	48	84	94	99
3	0.03	61	91	93	96
4	0.05	91	99	97	99
5	0.08	100	100	100	100
6	0.01	9	23	46	77
7	0.05	71	98	98	99
8	0.05	87	98	98	98
Average power (%)		59.88	75.75	82.00	91.13
Running time (Hour)		0.3142	1.1244	3.9969	39.5439

* In the third simulation study, there are four datasets consisting of 500, 1000, 2000, and 4000 individuals, respectively, and 10,000 SNP markers, with 100 replicates. Eight true QTNs are set in each replicate. Then, each dataset can be regarded as having 1,000,000 SNPs and 800 true QTNs in total.

**Table 4:** Previously reported genes that were identified at least by two methods simultaneously with HRePML, MLMM, FarmCPU, and GEMMA.

Detected Genes	Associated Trait	Chr.	Position	Effect Estimate	LOD/*p*-Value	Methods
*AT2G16440*	LFS GH	2	7140030	−7.461, −9.107, −5.16	$3.90\times 10^{-11},\;1.28\times 10^{-17},\;9.56\times 10^{-8}$	FarmCPU, MLMM, GEMMA
*AT3G07160*	LFS GH	3	2280271	−5.934, −8.845	$1.16\times 10^{-7},\;9.37\times 10^{-15}$	FarmCPU, MLMM
*AT3G54280*	MT GH	3	20090780	1.002, 1.762	$9.90\times 10^{-13},\;5.65\times 10^{-8\;}$	FarmCPU, MLMM
*AT4G09960*	FT Duration GH	4	6228754	0.822, 1.136	3.74, $3.69\times 10^{-8}$	HRePML, FarmCPU
*AT4G33620*	LC Duration GH	4	16140068	2.996, 2.540	4.78, $4.29\times 10^{-29}$	HRePML, MLMM
*AT5G45900,* *AT5G45940*	LC Duration GH	5	18625634, 18625726	−3.707, −6.051	4.78, $2.51\times 10^{-28}$	HRePML, FarmCPU
*AT5G45900,* *AT5G45940*	LFS GH	5	18625634, 18625726, 18625726	−4.318, −5.147, −5.616	5.23, $1.83\times 10^{-8},\;1.05\times 10^{-7}$	HRePML, FarmCPU, GEMMA
*AT5G53360*	MT GH	5	21646741	0.236, 0.267	$3.05\times 10^{-14},\;1.55\times 10^{-7}$	FarmCPU, GEMMA

## Supplementary Materials

The following are available online at https://www.mdpi.com/2073-4425/11/11/1286/s1, Table S1: Comparison of statistical power and mean squared errors (MSE) for each QTN among HRePML, MLMM, FarmCPU and GEMMA methods in the second simulation study, Table S2: Comparison of average statistical power, average mean squared errors (MSE) and running time among HRePML, MLMM, FarmCPU and GEMMA methods in the second simulation study, Table S3: Parameters settings including true effect for each QTN with different sample size in the third simulation study and true effect for each QTN with different number of markers in the fourth simulation study, Table S4: The numbers of SNPs significantly associated with four development related traits in Arabidopsis thaliana and the number of genes around these SNPs identified by HRePML, MLMM, FarmCPU and GEMMA methods, Table S5: GWAS for four development related traits in *Arabidopsis thaliana* using HRePML, MLMM, FarmCPU and GEMMA methods.
