# Supplementary material for: Hybrid of Restricted and Penalized Maximum Likelihood Method for Efficient Genome-Wide Association Study
Source: Genes (Basel). 2020 Oct 29;11(11):1286. doi: 10.3390/genes11111286 (PMC7692801; doi:10.3390/genes11111286)
Supplement: Supplementary file 1 [file genes-11-01286-s001.pdf]

Hybrid of Restricted and Penalized Maximum Likelihood Method for Efficient Genome-wide Association Study

Wenlong Ren [1], Zhikai Liang [2], Shu He [1], Jing Xiao [1,*]

[1] Department of Epidemiology and Medical Statistics, School of Public Health, Nantong University, Nantong, Jiangsu, China

[2] Plant and Microbial Biology Department, University of Minnesota, Saint Paul, USA

*** Correspondence:**
Jing Xiao
jxiaont@ntu.edu.cn

Supplementary Material

Table S1. Comparison of statistical power and mean squared errors (MSE) for each QTN among HRePML, MLMM, FarmCPU and GEMMA methods in the second simulation study*.

QTN	Chr.	Position(bp)	R^2	Effect	Power (%)				Mean Squared Errors (MSE)			
					HRePML	MLMM	FarmCPU	GEMMA	HRePML	MLMM	FarmCPU	GEMMA
1	1	404108	0.01	0.4742	12.1	3.4	2.9	0.0	0.0633	0.1841	0.1055	-
2	1	636788	0.03	0.8213	46.3	54.1	64.7	0.7	0.0311	0.0473	0.0342	0.2322
3	3	507976	0.03	0.8213	71.1	46.0	18.4	10.1	0.1926	0.1124	0.1223	0.1719
4	3	931437	0.05	1.0603	88.7	63.5	47.6	43.5	0.0528	0.0265	0.0954	0.0366
5	4	75898	0.08	1.3412	100.0	99.5	100.0	99.4	0.0490	0.0381	0.0619	0.0345
6	4	461978	0.01	0.4742	10.1	6.0	10.4	1.0	0.2273	0.4381	0.3674	0.6338
7	4	607026	0.05	1.0603	74.8	86.9	99.3	92.3	0.0856	0.1720	0.1277	0.2630
8	5	282008	0.05	1.0603	96.5	97.2	98.1	76.7	0.0390	0.0561	0.0331	0.1158

* In the second simulation study, the data set consists of 500 individuals and 10,000 SNP markers with 1,000 replicates. And 8 true QTNs are set in each replicate. Then, this dataset can be regarded as having 10,000,000 SNPs and 8,000 true QTNs in total.

Table S2. Comparison of average statistical power, average mean squared errors (MSE) and running time among HRePML, MLMM, FarmCPU and GEMMA methods in the second simulation study*.

Statistical Properties	HRePML	MLMM	FarmCPU	GEMMA
Average power (%)	62.45	57.08	55.18	40.46
Average MSE	0.0926	0.1343	0.1184	0.2125
Running time (Hour)	3.2273	27.4473	4.9198	2.3855

* The data set used in Table S2 are the same with those used in Table S1.

Table S3. Parameters settings including true effect for each QTN with different sample size in the third simulation study* and true effect for each QTN with different number of markers in the fourth simulation study#.

QTN	Chr.	Position(bp)	R^2	Sample Size: Effect				Number of Markers: Effect			
				500	1000	2000	4000	10000	50000	100000	200000
1	1	404108	0.01	0.4742	0.4340	0.4334	0.4317	0.4742	0.4239	0.4496	0.4551
2	1	636788	0.03	0.8213	0.7584	0.7682	0.7745	0.8213	0.8094	0.8305	0.7479
3	3	507976	0.03	0.8213	0.7526	0.7409	0.7452	0.8213	0.7359	0.7266	0.7554
4	3	931437	0.05	1.0603	0.9883	0.9691	0.9665	1.0603	0.9399	1.0053	0.9856
5	4	75898	0.08	1.3412	1.2113	1.2192	1.2195	1.3412	1.2214	1.2606	1.2071
6	4	461978	0.01	0.4742	0.4263	0.4303	0.4286	0.4742	0.4384	0.4278	0.4195
7	4	607026	0.05	1.0603	0.9816	0.9869	1.0005	1.0603	1.0024	1.0145	0.9966
8	5	282008	0.05	1.0603	0.9691	0.9772	0.9743	1.0603	0.9610	0.9522	1.0145

* In the third simulation study, there are four data sets consisting of 500, 1,000, 2,000 and 4,000 individuals, respectively, and 10,000 SNP markers, with 100 replicates. And 8 true QTNs are set in each replicate. Then, each dataset can be regarded as having 1,000,000 SNPs and 800 true QTNs in total.

# In the fourth simulation study, there are four data sets consisting of 10,000, 50,000, 100,000 and 200,000 SNP markers, respectively, and 500 individuals, with 100 replicates. And 8 true QTNs are set in each replicate. Then, four data sets can be regarded as having 1,000,000, 5,000,000, 10,000,000 and 20,000,000 SNPs, respectively, and 800 true QTNs.

Table S4. The numbers of SNPs significantly associated with four development related traits in *Arabidopsis* thaliana and the number of genes around these SNPs identified by HRePML, MLMM, FarmCPU and GEMMA methods.

Trait	Number of SNPs Significantly Associated with Traits				Number of Known Genes Detected			
	HRePML	MLMM	FarmCPU	GEMMA	HRePML	MLMM	FarmCPU	GEMMA
FT Duration GH	10	2	5	7	6	1	1	1
LC Duration GH	26	31	11	2	11	13	7	0
LFS GH	23	7	6	5	13	4	4	3
MT GH	18	3	10	3	11	1	13	1
Total	77	43	32	17	41	19	25	5

FT Duration GH: number of days between appearance of the first flower and the senescence of the last flower, LC Duration GH: number of days between germination and plant complete senescence, LFS GH: number of days between germination and senescence of the last flower and MT GH: number of days between last flower senescence and complete plant senescence.

Table S5. GWAS for four development related traits in *Arabidopsis* thaliana using HRePML, MLMM, FarmCPU and GEMMA methods.

Detected Genes	Associated Trait	Chromosome	Position	Effect Estimate	LOD/*P*-value	Methods	Reference
AT1G01690	LFS GH	1	252095	-2.906	8.12	HRePML	[1]
AT1G06150	FT Duration GH	1	1876943	1.027	4.07	HRePML	[2]
AT1G06170	FT Duration GH	1	1876943	1.027	4.07	HRePML	[3]
AT1G08410	LC Duration GH	1	2653741	-2.707	7.85	HRePML	[4]
AT1G09100	LC Duration GH	1	2948670	-1.634	3.02	HRePML	[5]
AT1G10745	LC Duration GH	1	3580647	-1.239	9.09E-10	MLMM	[6]
AT1G10747	LC Duration GH	1	3580647	-1.239	9.09E-10	MLMM	[6]
AT1G13590	FT Duration GH	1	4653501	-2.752	7.96E-08	MLMM	[7]
AT1G14280	LC Duration GH	1	4882806	-6.469	9.29E-13	FarmCPU	[8]
AT1G14320	LC Duration GH	1	4882806	-6.469	9.29E-13	FarmCPU	[9]
AT1G14350	LFS GH	1	4912083	2.819	4.67	HRePML	[10]
AT1G14360	LFS GH	1	4912083	2.819	4.67	HRePML	[11]
AT1G18835	MT GH	1	6499060	0.448	4.08	HRePML	[12]
AT1G27135	MT GH	1	9427585	0.526	4.31	HRePML	-
AT1G30000	MT GH	1	10515519	-0.576	6.86	HRePML	[13]
AT1G30010	MT GH	1	10515519	-0.576	6.86	HRePML	[14]
AT1G47200	LC Duration GH	1	17306925	1.116	3.96E-10	MLMM	[15]
AT1G55460	MT GH	1	20709875	-0.298	3.34	HRePML	[16]
AT1G58210	LFS GH	1	21551184	2.483	4.29	HRePML	[17]
AT1G60370	FT Duration GH	1	22248393	-0.927	3.83	HRePML	[18]
AT1G62060	MT GH	1	22945740	0.419	3.26	HRePML	[19]
AT1G62080	MT GH	1	22945740	0.419	3.26	HRePML	-
AT1G62085	MT GH	1	22945740	0.419	3.26	HRePML	-
AT1G62430	LFS GH	1	23112452	-1.836	3.18	HRePML	[20]
AT1G62830	MT GH	1	23267151	-0.510	3.33	HRePML	[21]
AT1G65480	LC Duration GH	1	24341923	-1.873	4.37	HRePML	[22]
AT1G74500	LFS GH	1	28003216	-1.838	3.09	HRePML	[23]
AT2G13540	MT GH	2	5638874	0.412	3.94	HRePML	[24]
AT2G15570	MT GH	2	6787293	-1.041	1.74E-15	FarmCPU	[25]
AT2G16440	LC Duration GH	2	7140030	-5.979	6.54E-22	FarmCPU	[26]
AT2G16440	LFS GH	2	7140030	-7.461, -9.107, -5.16	3.90E-11, 1.28E-17, 9.56E-08	FarmCPU, MLMM, GEMMA	[26]

AT2G16500	LC Duration GH	2	7154976	1.644	3.39	HRePML	[27]
AT2G19690	LC Duration GH	2	8506413	1.213	3.47	HRePML	[28]
AT2G31070	LC Duration GH	2	13226944	3.434	10.91	HRePML	[29]
AT3G01530	LFS GH	3	212444	-4.805	1.22E-08	MLMM	[30]
AT3G03250	LC Duration GH	3	746520	1.947	3.96	HRePML	[31]
AT3G03250	LFS GH	3	746520	2.037	3.08	HRePML	[31]
AT3G07160	LFS GH	3	2280271	-5.934, -8.845	1.16E-07, 9.37E-15	FarmCPU, MLMM	[32]
AT3G22820	LC Duration GH	3	8066094	1.982	3.79	HRePML	[33]
AT3G22840	FT Duration GH	3	8086127	1.154	4.32	HRePML	[34]
AT3G28990	LC Duration GH	3	11006055	2.373	2.74E-24	MLMM	-
AT3G29030	LC Duration GH	3	11006055	2.373	2.74E-24	MLMM	[35]
AT3G47870	MT GH	3	17650250	0.446	3.74	HRePML	[36]
AT3G48610	LC Duration GH	3	18021733	-3.033	4.99E-23	MLMM	[37]
AT3G54280	MT GH	3	20090780	1.002, 1.762	9.90E-13, 5.65E-08	FarmCPU, MLMM	[38]
AT3G56640	LFS GH	3	20981902	1.908	3.23	HRePML	[39]
AT3G62500	LC Duration GH	3	23110632	2.381	1.23E-27	MLMM	-
AT4G00020	MT GH	4	15364	1.136	3.59E-14	FarmCPU	[40]
AT4G00026	MT GH	4	15364	1.136	3.59E-14	FarmCPU	[41]
AT4G00050	MT GH	4	15364	1.136	3.59E-14	FarmCPU	[42]
AT4G00060	MT GH	4	15364	1.136	3.59E-14	FarmCPU	[43]
AT4G00260	LFS GH	4	117376	-3.176	4.63	HRePML	-
AT4G01250	LC Duration GH	4	529409	-3.924	2.29E-14	FarmCPU	[44]
AT4G05410	LFS GH	4	2742918	5.024	9.97	HRePML	[45]
AT4G09960	FT Duration GH	4	6228754	0.822, 1.136	3.74, 3.69E-08	HRePML, FarmCPU	[46]
AT4G31120	LFS GH	4	15126597	-10.514	4.29E-16	MLMM	[47]
AT4G33240	FT Duration GH	4	16028697	1.061	4.47	HRePML	[48]
AT4G33620	LC Duration GH	4	16140068	2.996, 2.540	4.78, 4.29E-29	HRePML, MLMM	[49]
AT4G33620	LFS GH	4	16140068	3.402	5.32	HRePML	[49]
AT5G13690	MT GH	5	4429786	1.702	4.44E-14	FarmCPU	[50]
AT5G13710	MT GH	5	4429786	1.702	4.44E-14	FarmCPU	[51]
AT5G13750	MT GH	5	4429786	1.702	4.44E-14	FarmCPU	[52]
AT5G24240	MT GH	5	8242061	1.426	4.47E-17	FarmCPU	[53]
AT5G27720	LC Duration GH	5	9820126	4.593	1.09E-48	MLMM	[54]
AT5G27740	LC Duration GH	5	9820126	4.593	1.09E-48	MLMM	-

AT5G35600	LC Duration GH	5	13764717	2.627	3.62E-30	MLMM	[55]
AT5G45300	LC Duration GH	5	18355835	-2.236	1.05E-29	MLMM	[56]
AT5G45900, AT5G45940	LC Duration GH	5	18625634, 18625726	-3.707, -6.051	4.78, 2.51E-28	HRePML, FarmCPU	[57]; [58]
AT5G45900, AT5G45940	LFS GH	5	18625634, 18625726, 18625726	-4.318, -5.147, -5.616	5.23, 1.83E-08, 1.05E-07	HRePML, FarmCPU, GEMMA	[57]; [58]
AT5G53360	MT GH	5	21646741	0.236, 0.267	3.05E-14, 1.55E-07	FarmCPU, GEMMA	[59]
AT5G54180	LC Duration GH	5	21982990	-2.129	1.84E-30	MLMM	[60]
AT5G58010	LC Duration GH	5	23476715	-4.708	6.50E-21	FarmCPU	[61]
AT5G59510	FT Duration GH	5	23989818	3.430	4.63E-08	GEMMA	-

Reference

1. De Muyt, A.; Pereira, L.; Vezon, D.; Chelysheva, L.; Gendrot, G.; Chambon, A.; Laine-Choinard, S.; Pelletier, G.; Mercier, R.; Nogue, F., et al. A high throughput genetic screen identifies new early meiotic recombination functions in Arabidopsis thaliana. *PLoS Genet* **2009**, *5*, e1000654, doi:10.1371/journal.pgen.1000654.
2. Ohashi-Ito, K.; Matsukawa, M.; Fukuda, H. An atypical bHLH transcription factor regulates early xylem development downstream of auxin. *Plant Cell Physiol* **2013**, *54*, 398-405, doi:10.1093/pcp/pct013.
3. Cui, J.; You, C.; Zhu, E.; Huang, Q.; Ma, H.; Chang, F. Feedback Regulation of DYT1 by Interactions with Downstream bHLH Factors Promotes DYT1 Nuclear Localization and Anther Development. *Plant Cell* **2016**, *28*, 1078-1093, doi:10.1105/tpc.15.00986.
4. Zhao, H.; Lu, S.; Li, R.; Chen, T.; Zhang, H.; Cui, P.; Ding, F.; Liu, P.; Wang, G.; Xia, Y., et al. The Arabidopsis gene DIG6 encodes a large 60S subunit nuclear export GTPase 1 that is involved in ribosome biogenesis and affects multiple auxin-regulated development processes. *J Exp Bot* **2015**, *66*, 6863-6875, doi:10.1093/jxb/erv391.
5. Ueda, M.; Matsui, K.; Ishiguro, S.; Kato, T.; Tabata, S.; Kobayashi, M.; Seki, M.; Shinozaki, K.; Okada, K. Arabidopsis RPT2a encoding the 26S proteasome subunit is required for various aspects of root meristem maintenance, and regulates gametogenesis redundantly with its homolog, RPT2b. *Plant Cell Physiol* **2011**, *52*, 1628-1640, doi:10.1093/pcp/pcr093.
6. Costa, L.M.; Marshall, E.; Tesfaye, M.; Silverstein, K.A.; Mori, M.; Umetsu, Y.; Otterbach, S.L.; Papareddy, R.; Dickinson, H.G.; Boutiller, K., et al. Central cell-derived peptides regulate early embryo patterning in flowering plants. *Science* **2014**, *344*, 168-172, doi:10.1126/science.1243005.
7. Matsubayashi, Y.; Ogawa, M.; Kihara, H.; Niwa, M.; Sakagami, Y. Disruption and overexpression of Arabidopsis phytosulfokine receptor gene affects cellular longevity and potential for growth. *Plant Physiol* **2006**, *142*, 45-53, doi:10.1104/pp.106.081109.
8. de Carbonnel, M.; Davis, P.; Roelfsema, M.R.; Inoue, S.; Schepens, I.; Lariguet, P.; Geisler, M.; Shimazaki, K.; Hangarter, R.; Fankhauser, C. The Arabidopsis PHYTOCHROME KINASE SUBSTRATE2 protein is a phototropin signaling element that regulates leaf flattening and leaf positioning. *Plant Physiol* **2010**, *152*, 1391-1405, doi:10.1104/pp.109.150441.
9. Zorzatto, C.; Machado, J.P.; Lopes, K.V.; Nascimento, K.J.; Pereira, W.A.; Brustolini, O.J.; Reis, P.A.; Calil, I.P.; Deguchi, M.; Sachetto-Martins, G., et al. NIK1-mediated translation suppression functions as a plant antiviral immunity mechanism. *Nature* **2015**, *520*, 679-682, doi:10.1038/nature14171.
10. Lei, Q.; Lee, E.; Keerthisinghe, S.; Lai, L.; Li, M.; Lucas, J.R.; Wen, X.; Ren, X.; Sack, F.D. The FOUR LIPS and MYB88 transcription factor genes are widely expressed in Arabidopsis thaliana during development. *Am J Bot* **2015**, *102*, 1521-1528, doi:10.3732/ajb.1500056.
11. Reyes, F.; Leon, G.; Donoso, M.; Brandizzi, F.; Weber, A.P.; Orellana, A. The nucleotide sugar transporters AtUTr1 and AtUTr3 are required for the incorporation of UDP-glucose into the endoplasmic reticulum, are essential for pollen development and are needed for embryo sac progress in Arabidopsis thaliana. *Plant J* **2010**, *61*, 423-435, doi:10.1111/j.1365-313X.2009.04066.x.
12. Hu, W.; Feng, B.; Ma, H. Ectopic expression of the Arabidopsis MINI ZINC FINGER1 and MIF3 genes induces shoot meristems on leaf margins. *Plant Mol Biol* **2011**, *76*, 57-68, doi:10.1007/s11103-011-9768-y.
13. Huttner, S.; Veit, C.; Vavra, U.; Schoberer, J.; Liebminger, E.; Maresch, D.; Grass, J.; Altmann, F.; Mach, L.; Strasser, R. Arabidopsis Class I alpha-Mannosidases MNS4 and MNS5 Are Involved in Endoplasmic Reticulum-Associated Degradation of Misfolded Glycoproteins. *Plant Cell* **2014**, *26*, 1712-1728, doi:10.1105/tpc.114.123216.
14. Keren, I.; Tal, L.; des Francs-Small, C.C.; Araujo, W.L.; Shevtsov, S.; Shaya, F.; Fernie, A.R.; Small, I.; Ostersetzer-Biran, O. nMAT1, a nuclear-encoded maturase involved in the trans-splicing of nad1 intron 1, is essential for mitochondrial complex I assembly and function. *Plant J* **2012**, *71*, 413-426, doi:10.1111/j.1365-313X.2012.04998.x.
15. Patel, S.; Rose, A.; Meulia, T.; Dixit, R.; Cyr, R.J.; Meier, I. Arabidopsis WPP-domain proteins are developmentally associated with the nuclear envelope and promote cell division. *Plant Cell* **2004**, *16*, 3260-3273, doi:10.1105/tpc.104.026740.
16. Garcia-Molina, A.; Xing, S.; Huijser, P. A conserved KIN17 curved DNA-binding domain protein assembles with SQUAMOSA PROMOTER-BINDING PROTEIN-LIKE7 to adapt Arabidopsis growth and development to limiting copper availability. *Plant Physiol* **2014**, *164*, 828-840, doi:10.1104/pp.113.228239.
17. Duckney, P.; Deeks, M.J.; Dixon, M.R.; Kroon, J.; Hawkins, T.J.; Hussey, P.J. Actin-membrane interactions mediated by NETWORKED2 in Arabidopsis pollen tubes through associations with Pollen Receptor-Like Kinase 4 and 5. *New Phytol* **2017**, *216*, 1170-1180, doi:10.1111/nph.14745.
18. Meijon, M.; Satbhai, S.B.; Tsuchimatsu, T.; Busch, W. Genome-wide association study using cellular traits identifies a new regulator of root development in Arabidopsis. *Nat Genet* **2014**, *46*, 77-81, doi:10.1038/ng.2824.

19. McGee, R.; Dean, G.H.; Mansfield, S.D.; Haughn, G.W. Assessing the utility of seed coat-specific promoters to engineer cell wall polysaccharide composition of mucilage. *Plant Mol Biol* **2019**, *101*, 373-387, doi:10.1007/s11103-019-00909-8.

20. Zhou, Y.; Peisker, H.; Weth, A.; Baumgartner, W.; Dormann, P.; Frentzen, M. Extraplastidial cytidinediphosphate diacylglycerol synthase activity is required for vegetative development in Arabidopsis thaliana. *Plant J* **2013**, *75*, 867-879, doi:10.1111/tpj.12248.

21. Martignago, D.; Bernardini, B.; Polticelli, F.; Salvi, D.; Cona, A.; Angelini, R.; Tavladoraki, P. The Four FAD-Dependent Histone Demethylases of Arabidopsis Are Differently Involved in the Control of Flowering Time. *Front Plant Sci* **2019**, *10*, 669, doi:10.3389/fpls.2019.00669.

22. Yu, Y.; Qiao, L.; Chen, J.; Rong, Y.; Zhao, Y.; Cui, X.; Xu, J.; Hou, X.; Dong, C.H. Arabidopsis REM16 acts as a B3 domain transcription factor to promote flowering time via directly binding to the promoters of SOC1 and FT. *Plant J* **2020**, 10.1111/tpj.14807, doi:10.1111/tpj.14807.

23. Li, T.; Lei, W.; He, R.; Tang, X.; Han, J.; Zou, L.; Yin, Y.; Lin, H.; Zhang, D. Brassinosteroids regulate root meristem development by mediating BIN2-UPB1 module in Arabidopsis. *PLoS Genet* **2020**, *16*, e1008883, doi:10.1371/journal.pgen.1008883.

24. Daszkowska-Golec, A.; Wojnar, W.; Rosikiewicz, M.; Szarejko, I.; Maluszynski, M.; Szweykowska-Kulinska, Z.; Jarmolowski, A. Arabidopsis suppressor mutant of abh1 shows a new face of the already known players: ABH1 (CBP80) and ABI4-in response to ABA and abiotic stresses during seed germination. *Plant Mol Biol* **2013**, *81*, 189-209, doi:10.1007/s11103-012-9991-1.

25. Batushansky, A.; Kirma, M.; Grillich, N.; Pham, P.A.; Rentsch, D.; Galili, G.; Fernie, A.R.; Fait, A. The transporter GAT1 plays an important role in GABA-mediated carbon-nitrogen interactions in Arabidopsis. *Front Plant Sci* **2015**, *6*, 785, doi:10.3389/fpls.2015.00785.

26. Rizvi, I.; Choudhury, N.R.; Tuteja, N. Arabidopsis thaliana MCM3 single subunit of MCM2-7 complex functions as 3' to 5' DNA helicase. *Protoplasma* **2016**, *253*, 467-475, doi:10.1007/s00709-015-0825-2.

27. El Amrani, A.; Couee, I.; Berthome, R.; Ramel, F.; Gouesbet, G.; Sulmon, C. Involvement of polyamines in sucrose-induced tolerance to atrazine-mediated chemical stress in Arabidopsis thaliana. *J Plant Physiol* **2019**, *238*, 1-11, doi:10.1016/j.jplph.2019.04.012.

28. Kim, H.J.; Ok, S.H.; Bahn, S.C.; Jang, J.; Oh, S.A.; Park, S.K.; Twell, D.; Ryu, S.B.; Shin, J.S. Endoplasmic reticulum- and Golgi-localized phospholipase A2 plays critical roles in Arabidopsis pollen development and germination. *Plant Cell* **2011**, *23*, 94-110, doi:10.1105/tpc.110.074799.

29. Bresso, E.G.; Chorostecki, U.; Rodriguez, R.E.; Palatnik, J.F.; Schommer, C. Spatial Control of Gene Expression by miR319-Regulated TCP Transcription Factors in Leaf Development. *Plant Physiol* **2018**, *176*, 1694-1708, doi:10.1104/pp.17.00823.

30. Bender, R.L.; Fekete, M.L.; Klinkenberg, P.M.; Hampton, M.; Bauer, B.; Malecha, M.; Lindgren, K.; J, A.M.; Perera, M.A.; Nikolau, B.J., et al. PIN6 is required for nectary auxin response and short stamen development. *Plant J* **2013**, *74*, 893-904, doi:10.1111/tpj.12184.

31. Fusari, C.M.; Kooke, R.; Lauxmann, M.A.; Annunziata, M.G.; Enke, B.; Hoehne, M.; Krohn, N.; Becker, F.F.M.; Schlereth, A.; Sulpice, R., et al. Genome-Wide Association Mapping Reveals That Specific and Pleiotropic Regulatory Mechanisms Fine-Tune Central Metabolism and Growth in Arabidopsis. *Plant Cell* **2017**, *29*, 2349-2373, doi:10.1105/tpc.17.00232.

32. Toller, A.; Brownfield, L.; Neu, C.; Twell, D.; Schulze-Lefert, P. Dual function of Arabidopsis glucan synthase-like genes GSL8 and GSL10 in male gametophyte development and plant growth. *Plant J* **2008**, *54*, 911-923, doi:10.1111/j.1365-313X.2008.03462.x.

33. Niwa, T.; Kondo, T.; Nishizawa, M.; Kajita, R.; Kakimoto, T.; Ishiguro, S. EPIDERMAL PATTERNING FACTOR LIKE5 peptide represses stomatal development by inhibiting meristemoid maintenance in Arabidopsis thaliana. *Biosci Biotechnol Biochem* **2013**, *77*, 1287-1295, doi:10.1271/bbb.130145.

34. Sun, T.; Zhou, F.; Huang, X.Q.; Chen, W.C.; Kong, M.J.; Zhou, C.F.; Zhuang, Z.; Li, L.; Lu, S. ORANGE Represses Chloroplast Biogenesis in Etiolated Arabidopsis Cotyledons via Interaction with TCP14. *Plant Cell* **2019**, *31*, 2996-3014, doi:10.1105/tpc.18.00290.

35. Nardi, C.F.; Villarreal, N.M.; Rossi, F.R.; Martinez, S.; Martinez, G.A.; Civello, P.M. Overexpression of the carbohydrate binding module of strawberry expansin2 in Arabidopsis thaliana modifies plant growth and cell wall metabolism. *Plant Mol Biol* **2015**, *88*, 101-117, doi:10.1007/s11103-015-0311-4.

36. Kim, M.J.; Kim, M.; Lee, M.R.; Park, S.K.; Kim, J. LATERAL ORGAN BOUNDARIES DOMAIN (LBD)10 interacts with SIDECAR POLLEN/LBD27 to control pollen development in Arabidopsis. *Plant J* **2015**, *81*, 794-809, doi:10.1111/tpj.12767.

37. Ngo, A.H.; Lin, Y.C.; Liu, Y.C.; Gutbrod, K.; Peisker, H.; Dormann, P.; Nakamura, Y. A pair of nonspecific phospholipases C, NPC2 and NPC6, are involved in gametophyte development and glycerolipid metabolism in Arabidopsis. *New Phytol* **2018**, *219*, 163-175, doi:10.1111/nph.15147.

38. Tamaki, H.; Konishi, M.; Daimon, Y.; Aida, M.; Tasaka, M.; Sugiyama, M. Identification of novel meristem factors involved in shoot regeneration through the analysis of temperature-sensitive mutants of Arabidopsis. *Plant J* **2009**, *57*, 1027-1039, doi:10.1111/j.1365-313X.2008.03750.x.

39. Beuder, S.; Dorchak, A.; Bhide, A.; Moeller, S.R.; Petersen, B.L.; MacAlister, C.A. Exocyst mutants suppress pollen tube growth and cell wall structural defects of hydroxyproline O-arabinosyltransferase mutants. *Plant J* **2020**, 10.1111/tpj.14808, doi:10.1111/tpj.14808.

40. Seeliger, K.; Dukowic-Schulze, S.; Wurz-Wildersinn, R.; Pacher, M.; Puchta, H. BRCA2 is a mediator of RAD51- and DMC1-facilitated homologous recombination in Arabidopsis thaliana. *New Phytol* **2012**, *193*, 364-375, doi:10.1111/j.1469-8137.2011.03947.x.

41. Hamasaki, H.; Yoshizumi, T.; Takahashi, N.; Higuchi, M.; Kuromori, T.; Imura, Y.; Shimada, H.; Matsui, M. SD3, an Arabidopsis thaliana homolog of TIM21, affects intracellular ATP levels and seedling development. *Mol Plant* **2012**, *5*, 461-471, doi:10.1093/mp/ssr088.

42. Oh, J.; Park, E.; Song, K.; Bae, G.; Choi, G. PHYTOCHROME INTERACTING FACTOR8 Inhibits Phytochrome A-Mediated Far-Red Light Responses in Arabidopsis. *Plant Cell* **2020**, *32*, 186-205, doi:10.1105/tpc.19.00515.

43. Lange, H.; Sement, F.M.; Canaday, J.; Gagliardi, D. Polyadenylation-assisted RNA degradation processes in plants. *Trends Plant Sci* **2009**, *14*, 497-504, doi:10.1016/j.tplants.2009.06.007.

44. Chung, B.Y.W.; Balcerowicz, M.; Di Antonio, M.; Jaeger, K.E.; Geng, F.; Franaszek, K.; Marriott, P.; Brierley, I.; Firth, A.E.; Wigge, P.A. An RNA thermoswitch regulates daytime growth in Arabidopsis. *Nat Plants* **2020**, *6*, 522-532, doi:10.1038/s41477-020-0633-3.

45. Li, H.J.; Liu, N.Y.; Shi, D.Q.; Liu, J.; Yang, W.C. YAO is a nucleolar WD40-repeat protein critical for embryogenesis and gametogenesis in Arabidopsis. *BMC Plant Biol* **2010**, *10*, 169, doi:10.1186/1471-2229-10-169.

46. Di Marzo, M.; Roig-Villanova, I.; Zanchetti, E.; Caselli, F.; Gregis, V.; Bardetti, P.; Chiara, M.; Guazzotti, A.; Caporali, E.; Mendes, M.A., et al. MADS-Box and bHLH Transcription Factors Coordinate Transmitting Tract Development in Arabidopsis thaliana. *Front Plant Sci* **2020**, *11*, 526, doi:10.3389/fpls.2020.00526.

47. Hu, P.; Zhao, H.; Zhu, P.; Xiao, Y.; Miao, W.; Wang, Y.; Jin, H. Dual regulation of Arabidopsis AGO2 by arginine methylation. *Nat Commun* **2019**, *10*, 844, doi:10.1038/s41467-019-08787-w.

48. Whitley, P.; Hinz, S.; Doughty, J. Arabidopsis FAB1/PIKfyve proteins are essential for development of viable pollen. *Plant Physiol* **2009**, *151*, 1812-1822, doi:10.1104/pp.109.146159.

49. Liu, L.; Jiang, Y.; Zhang, X.; Wang, X.; Wang, Y.; Han, Y.; Coupland, G.; Jin, J.B.; Searle, I.; Fu, Y.F., et al. Two SUMO Proteases SUMO PROTEASE RELATED TO FERTILITY1 and 2 Are Required for Fertility in Arabidopsis. *Plant Physiol* **2017**, *175*, 1703-1719, doi:10.1104/pp.17.00021.

50. Ronceret, A.; Gadea-Vacas, J.; Guilleminot, J.; Devic, M. The alpha-N-acetyl-glucosaminidase gene is transcriptionally activated in male and female gametes prior to fertilization and is essential for seed development in Arabidopsis. *J Exp Bot* **2008**, *59*, 3649-3659, doi:10.1093/jxb/ern215.

51. Nakamoto, M.; Schmit, A.C.; Heintz, D.; Schaller, H.; Ohta, D. Diversification of sterol methyltransferase enzymes in plants and a role for beta-sitosterol in oriented cell plate formation and polarized growth. *Plant J* **2015**, *84*, 860-874, doi:10.1111/tpj.13043.

52. Remy, E.; Cabrito, T.R.; Baster, P.; Batista, R.A.; Teixeira, M.C.; Friml, J.; Sa-Correia, I.; Duque, P. A major facilitator superfamily transporter plays a dual role in polar auxin transport and drought stress tolerance in Arabidopsis. *Plant Cell* **2013**, *25*, 901-926, doi:10.1105/tpc.113.110353.

53. Akhter, S.; Uddin, M.N.; Jeong, I.S.; Kim, D.W.; Liu, X.M.; Bahk, J.D. Role of Arabidopsis AtPI4Kgamma3, a type II phosphoinositide 4-kinase, in abiotic stress responses and floral transition. *Plant Biotechnol J* **2016**, *14*, 215-230, doi:10.1111/pbi.12376.

54. Zhang, Z.; Zhang, S.; Zhang, Y.; Wang, X.; Li, D.; Li, Q.; Yue, M.; Li, Q.; Zhang, Y.E.; Xu, Y., et al. Arabidopsis floral initiator SKB1 confers high salt tolerance by regulating transcription and pre-mRNA splicing through altering histone H4R3 and small nuclear ribonucleoprotein LSM4 methylation. *Plant Cell* **2011**, *23*, 396-411, doi:10.1105/tpc.110.081356.

55. Cigliano, R.A.; Cremona, G.; Paparo, R.; Termolino, P.; Perrella, G.; Gutzat, R.; Consiglio, M.F.; Conicella, C. Histone deacetylase AtHDA7 is required for female gametophyte and embryo development in Arabidopsis. *Plant Physiol* **2013**, *163*, 431-440, doi:10.1104/pp.113.221713.

56. Soyk, S.; Simkova, K.; Zurcher, E.; Luginbuhl, L.; Brand, L.H.; Vaughan, C.K.; Wanke, D.; Zeeman, S.C. The Enzyme-Like Domain of Arabidopsis Nuclear beta-Amylases Is Critical for DNA Sequence Recognition and Transcriptional Activation. *Plant Cell* **2014**, *26*, 1746-1763, doi:10.1105/tpc.114.123703.

57. Barros, J.A.S.; Cavalcanti, J.H.F.; Medeiros, D.B.; Nunes-Nesi, A.; Avin-Wittenberg, T.; Fernie, A.R.; Araujo, W.L. Autophagy Deficiency Compromises Alternative Pathways of Respiration following Energy Deprivation in Arabidopsis thaliana. *Plant Physiol* **2017**, *175*, 62-76, doi:10.1104/pp.16.01576.

58. Ito, D.; Yoshimura, K.; Ishikawa, K.; Ogawa, T.; Maruta, T.; Shigeoka, S. A comparative analysis of the molecular characteristics of the Arabidopsis CoA pyrophosphohydrolases AtNUDX11, 15, and 15a. *Biosci Biotechnol Biochem* **2012**, *76*, 139-147, doi:10.1271/bbb.110636.

59. Qi, H.; Li, J.; Xia, F.N.; Chen, J.Y.; Lei, X.; Han, M.Q.; Xie, L.J.; Zhou, Q.M.; Xiao, S. Arabidopsis SINAT Proteins Control Autophagy by Mediating Ubiquitylation and Degradation of ATG13. *Plant Cell* **2020**, *32*, 263-284, doi:10.1105/tpc.19.00413.

60. Xiong, H.B.; Wang, J.; Huang, C.; Rochaix, J.D.; Lin, F.M.; Zhang, J.X.; Ye, L.S.; Shi, X.H.; Yu, Q.B.; Yang, Z.N. mTERF8, a Member of the Mitochondrial Transcription Termination Factor Family, Is Involved in the Transcription Termination of Chloroplast Gene psbJ. *Plant Physiol* **2020**, *182*, 408-423, doi:10.1104/pp.19.00906.

61. Tam, T.H.; Catarino, B.; Dolan, L. Conserved regulatory mechanism controls the development of cells with rooting functions in land plants. *Proc Natl Acad Sci U S A* **2015**, *112*, E3959-3968, doi:10.1073/pnas.1416324112.
